# A trade-off between thickness and length in the zebra finch sperm mid-piece

**DOI:** 10.1098/rspb.2018.0865

**Published:** 2018-07-25

**Authors:** Tania Mendonca, Tim R. Birkhead, Ashley J. Cadby, Wolfgang Forstmeier, Nicola Hemmings

**Affiliations:** 1 Department of Animal and Plant Sciences, University of Sheffield, Western Bank, Sheffield S10 2TN, UK; 2 Department of Physics and Astronomy, University of Sheffield, Western Bank, Sheffield S10 2TN, UK; 3 Department of Behavioural Ecology and Evolutionary Genetics, Max Planck Institute for Ornithology, Eberhard-Gwinner-Straße, 82319 Seewiesen, Germany

**Keywords:** zebra finch, sperm morphology, mitochondrial helix, mid-piece volume, ATP, cristae density

## Abstract

The sperm mid-piece has traditionally been considered to be the engine that powers sperm. Larger mid-pieces have therefore been assumed to provide greater energetic capacity. However, in the zebra finch *Taeniopygia guttata*, a recent study showed a surprising negative relationship between mid-piece length and sperm energy content. Using a multi-dimensional approach to study mid-piece structure, we tested whether this unexpected relationship can be explained by a trade-off between mid-piece length and mid-piece thickness and/or cristae density inside the mitochondrial helix. We used selective plane illumination microscopy to study mid-piece structure from three-dimensional images of zebra finch sperm and used high-resolution transmission electron microscopy to quantify mitochondrial density. Contrary to the assumption that longer mid-pieces are larger and therefore produce or contain a greater amount of energy, our results indicate that the amount of mitochondrial material is consistent across mid-pieces of varying lengths, and longer mid-pieces are simply proportionately ‘thinner’.

## 1. Introduction

Sperm are one of the most morphologically diverse cell types in internal fertilizers, evolving in response to strong selection within the female reproductive tract [1,2]. To reach and fertilize the ovum, sperm must travel through the challenging environment of the female tract, often competing with sperm from other males [3]. Morphological traits that confer enhanced motility and/or higher energetic capacity should therefore be favoured.

Studies in a range of taxa have demonstrated that species with high sperm competition intensity produce sperm that are longer [4,5] (but see [6,7]), faster [8–10] and show greater morphological uniformity [11–16]. There is limited but contradictory information on the association between sperm competition intensity and sperm energetics; Tourmente *et al.* [17] found a positive relationship between sperm competition intensity and levels of adenosine triphosphate (ATP), a crucial nucleotide for metabolism, across the sperm of nine rodent species, but Rowe *et al.* [18] found no such relationship across 23 passerine birds. Rowe *et al.* [18] also found that intracellular sperm ATP concentration was positively related to sperm mid-piece length, suggesting that ATP is generated primarily through oxidative phosphorylation (OXPHOS) in the mitochondria within the sperm mid-piece in these species. However, in a model passerine, the zebra finch *Taeniopygia guttata*, sperm with short mid-pieces have been shown to contain higher concentrations of stored ATP than those with long mid-pieces [19]. This is surprising, because it is reasonable to assume that larger mid-pieces contain more mitochondrial material, resulting in greater ATP production.

It is possible that ATP production via OXPHOS in the mid-piece is supplemented or replaced by another metabolic pathway, glycolysis, which primarily occurs in the anaerobic environment of the cytosol in sperm flagella, as appears to be the case in some mammalian sperm [20]. However, in the mammals studied, glycolytic enzymes are reportedly bound to the fibrous sheath present along the principal piece of the flagellum [21], and such a fibrous sheath does not appear to be present in passerine sperm [22]. Moreover, because OXPHOS has been shown to be a major energetic pathway for other (non-passerine) bird sperm [23,24], current evidence suggests that mitochondrial activity is likely to be important for passerine sperm energetics.

Assuming mitochondrial activity is important for zebra finch sperm motility, the disparity between mid-piece length and stored ATP content may be interpreted in two ways, which are not mutually exclusive: (i) length alone may not be an accurate measurement of mid-piece size (i.e. mid-piece volume may not be proportional to length), and/or (ii) the internal organization of the fused mitochondria inside the mid-piece may allow for greater energetic efficiency in shorter mid-pieces. In mitochondria, the majority of the major protein complexes involved in OXPHOS metabolism [25] are localized in membrane folds called cristae scattered among the mitochondrial matrix. Mitochondria are dynamic structures with cristae packing density increasing with energy demand to form a more interconnected topology [26], including within a cell type [27]. Increased cristae density improves the flow of adenylates to metabolic sites, increasing ATP production [28]. This suggests that mitochondrial packing, along with overall mitochondrial volume, is important for mitochondrial activity.

A small number of mammalian studies have made volumetric measurements of the sperm mid-piece [6,29,30], but this approach has not yet been extended to other taxa. Obtaining volumetric data from passerine sperm is particularly challenging, due to their helical shape [22]. Here, we overcame these challenges by employing three-dimensional imaging techniques to obtain volumetric data from zebra finch sperm, combined with transmission electron microscopy (TEM) to reveal sperm mid-piece internal structure. Our aims were (i) to determine the relationship between sperm mid-piece volume and length, and (ii) to assess whether mitochondrial organization inside the mid-piece varies with mid-piece length.

## 2. Material and methods

Our study had two main parts; the first investigated the structure of the sperm mitochondrial helix in zebra finch sperm, using selective plane illumination microscopy (SPIM), a fluorescence-based 3D imaging technique. The second examined the internal structure of the zebra finch sperm mitochondrial helix using TEM.

### (a) Part 1: mid-piece structure

#### (i) Animals

For part 1, zebra finches were from a domesticated population at the University of Sheffield that had been subjected to a selective breeding regime to increase numbers of males producing sperm at the extreme ends of the species's total sperm length range [31]. Sperm length data were routinely collected from faecal sperm samples as described by Immler & Birkhead [32]. Using these data, we selected 10 males producing sperm with relatively long mid-pieces (mean ± s.d.: 37.209 ± 2.623 µm), and 10 with relatively short mid-pieces (mean ± s.d.: 26.822 ± 4.447 µm), all hatched within the same year (within male repeatability *R* = 0.78 [33]). Sperm from one of the short mid-piece males was not included in the final analysis because it showed a high proportion of structurally damaged sperm (e.g. broken/missing sperm cell components).

Birds were housed in cages (dimensions: 1.2 × 0.5 × 0.4 m) in groups of 10, separated from groups of 10 females in adjacent identical cages behind a wire mesh. This arrangement kept males physically separated from the females (to avoid sperm depletion [34]) while still allowing them to receive visual and acoustic cues to stimulate sperm production. Prior to sperm collection, males were humanely euthanized by cervical dislocation in accordance with Schedule 1 (Animals (Scientific Procedures) Act 1986) and dissected immediately. Semen was collected from the left seminal glomerus (SG) by squeezing the distal region into warm Ham's F10 Nutrient Mix (Life Technologies, UK) as described by Bennison *et al.* [31]. Twenty microlitres of Ham's F10 media containing motile sperm from the SG were collected under a dissection microscope and fixed in 300 µl of 5% (v/v) formalin.

#### (ii) Microscopy

Ten microlitres aliquots of the fixed sperm samples from each male (*n* = 19 males) were labelled with 500 µmol l^−1^ MitoTracker Green FM (Molecular Probes, Eugene, OR, USA), and then imaged using a custom-built SPIM microscope with resolution comparable to that of widefield fluorescence microscopes [35] to acquire three-dimensional image stacks of sperm.

**Figure 1.**
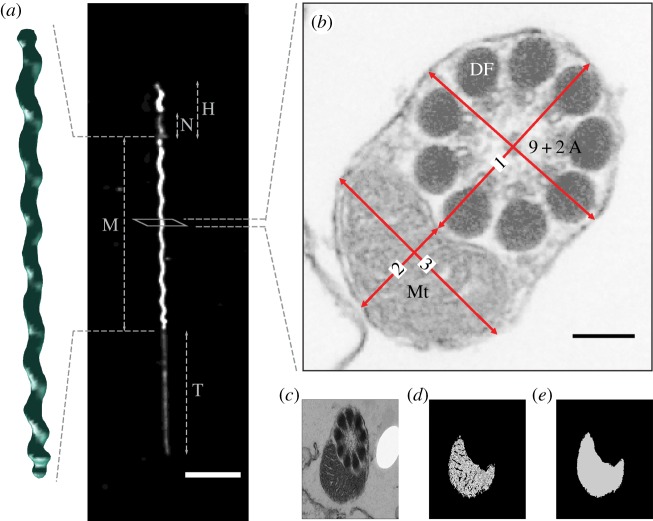
(*a*) A typical zebra finch sperm consists of a head ‘H’ (which includes the nucleus ‘N’), mid-piece ‘M’ and tail ‘T’. Scale bar, 10 µm. Volumetric measures of mitochondrial helices were acquired from three-dimensional images taken with SPIM in part 1. (*b*) Sections through the mid-piece were imaged using TEM in part 2, showing mitochondria (Mt), and the 9 + 2 axonemal structure (9 + 2 A) and electron-dense bodies called dense fibres (DF) within the flagellum. Measurements were made for (1) flagellum diameter, and mitochondrial (2) minor and (3) major axis diameters. Scale bar, 100 nm. Cristae packing was measured from sperm mid-piece cross-sections; (*c*) original TEM image, (*d*) area occupied by mitochondrial matrix and (*e*) area occupied by mitochondria.

The length and volume of the mid-piece of individual sperm were computed from image stacks (figure 1*a*; electronic supplementary material) and the number of gyres (helical turns) along each mid-piece was recorded. Immature and damaged sperm (with kinks, breaks or swellings) were excluded from analysis. After these exclusions, a total of five sperm from 11 males, four sperm from seven males and three sperm from one male were processed in this way.

### (b) Part 2: mid-piece internal organization

#### (i) Animals

For part 2, zebra finches were from the captive study population ‘Bielefeld’ held at the Max Planck Institute for Ornithology in Seewiesen [36]. Birds were housed in large semi-outdoor aviaries (dimensions: 5 × 2 × 2.5 m), where they had been breeding and raising offspring (approx. six breeding pairs per aviary) until 68 days before collection. During the post-breeding episode of 68 days, 27 males were housed in one unisex aviary. Ten of these males (age range 3.4–3.6) were used for this study. These birds had been genotyped for the Z-chromosome inversion that is known to affect mid-piece length in zebra finches [37]. The homokaryotype AA (ancestral allele) has been shown to determine the production of sperm with the shortest mid-piece lengths, while the heterokaryotype AB is associated with long mid-piece lengths [37,38]. Five males with the AA genotype and five with the AB genotype were selected randomly (without information on their sperm morphology) for use. The birds were euthanized by cervical dislocation and dissected immediately. The left SG of each male was immediately removed and the distal 3–4 mm of the SG was unravelled. A 10 µl sample of sperm was collected as described in part 1, except that sperm were released into phosphate-buffered saline (PBS, Sigma Aldrich) instead of Ham's F12 nutrient media. This sperm sample was fixed in 200 µl of 5% (v/v) formalin and used to measure the lengths of the sperm components. The rest of the SG was prepared for TEM (see electronic supplementary material, methods for details).

#### (ii) Microscopy

Since sperm were sliced to obtain transverse sections, mid-piece lengths could not be obtained from the TEM images. Therefore, samples of fixed sperm (see above) from the same males (*n* = 10 males, 10 sperm from each) were analysed to acquire the mean mid-piece lengths for each male (within male repeatability *R* = 0.84 [33]) as in Bennison *et al.* [31].

The density of cristae in the mitochondria was measured from the TEM images as the proportion of the area of mitochondria occupied by cristae (*n* = 10 males, 20 measures from each; figure 1*c*). From the high-resolution TEM sperm cross sections, it was also possible to obtain accurate mid-piece diameter measurements (figure 1*b*). Mitochondrial cross-sectional area was computed using the formula for the area of an ellipse (*π* × *a* × *b*; where *a* and *b* are the mitochondrial major and minor axis radii, respectively, figure 1*b*). A limitation of the TEM technique is that cross sections of individual sperm cannot be distinguished and although care was taken not to measure cross sections from the same location in consecutive images, we do not know how many individual sperm were measured in total per male.

### (c) Statistical analysis

Statistical analysis was performed using R v. 3.2.3 [39]. A mixed effects model was parameterized using the ‘lmer’ function from the ‘lme4’ [40] and the ‘lmerTest’ [41] packages, to test the effect of mid-piece length on number of helical gyres, both measured from SPIM images in part 1. This model included the count of gyres along the mid-piece as the dependent variable, mid-piece length as a fixed effect and bird ID as a random effect. A mid-piece of zero length has zero gyres by definition, so the intercept was removed from this model.

Using measurements from TEM images in part 2 of the study, three more mixed effects models were computed as above using the ‘lmer’ function. The first model assessed the effect of mid-piece length on the cross-sectional area of the mitochondria and included the cross-sectional area of the mitochondria as a dependent variable. The second was defined as a quadratic model with an index of cristae density (computed as the proportion of mitochondria occupied by cristae) as a dependent variable. An additional model was computed to test the effect of mid-piece length on the radius of the mid-piece helix (half the sum of measures ‘1’ and ‘2’ from figure 1*b*). The above models included mid-piece length as an explanatory variable and bird ID as a random effect. Log transformation was applied to the data in the first model to compute the contrast of the regression slope to a slope of ‘0’ (i.e. constant area) or ‘−1’ (i.e. constant volume) using Student's *t* distribution.

## 3. Results

### (a) Part 1: mid-piece structure

**Figure 2.**
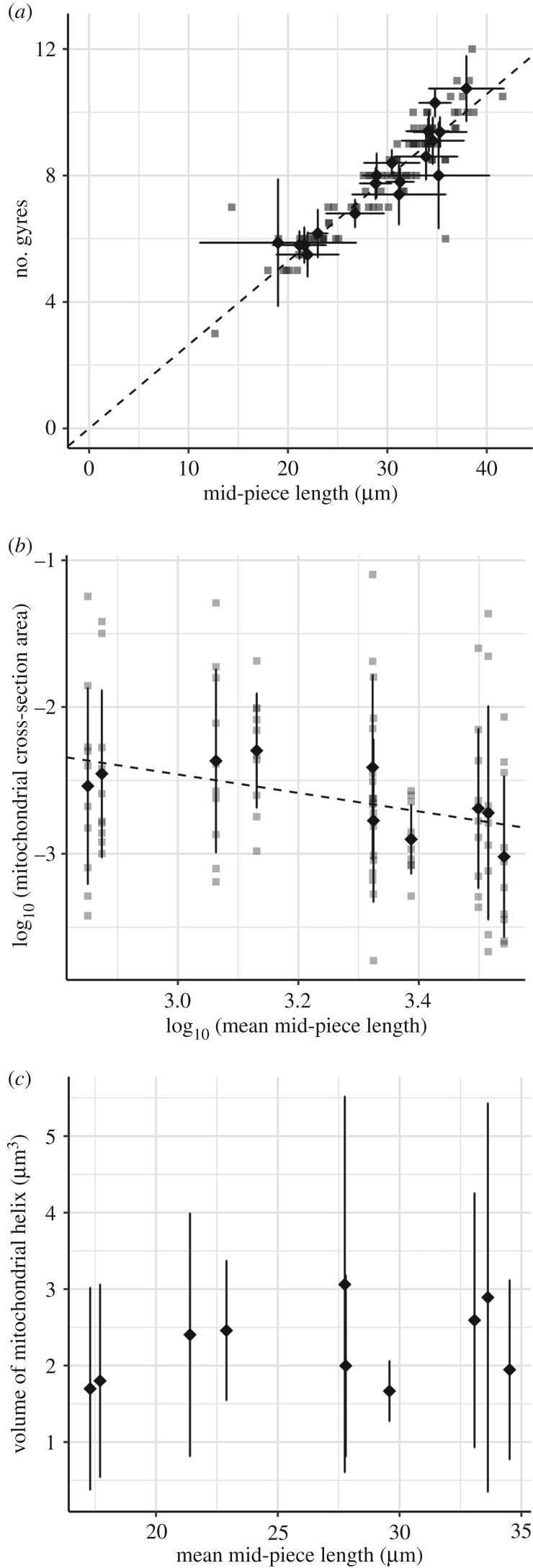
Effect of mid-piece length on (*a*) number of helical gyres (*n* = 19 males, 87 sperm) in part 1 and (*b*) mitochondrial cross-section area and (*c*) mitochondrial helix volume (*n* = 10 males, 10 measures each) in part 2. Volumes were computed from mitochondrial cross-section areas and mean straightened helical length for each male. Grey data points represent individual sperm, diamond-shaped points represent mean values and the error bars represent the standard deviation for each male.

The average diameter of the zebra finch sperm mid-piece (see Part 2) was close to the resolution limit of the SPIM (electronic supplementary material). This meant that the technique lacked the sensitivity to capture the relationship between mid-piece volume and length. The three-dimensional SPIM images did, however, provide accurate length measurements for sperm. From these measurements, mid-piece length was found to be highly correlated with the number of gyres along the mitochondrial helix, such that an increase in length corresponded with an increase in the number of gyres (estimated effect = 0.264 gyres µm**^−^**^1^, *t* = 61.7, *p* ≤ 0.0001; figure 2*a*; *n* = 19 males). This defines a mid-piece structure with a consistent gyre height of 3.783 µm (1/0.264).

### (b) Part 2: mid-piece internal organization

Mitochondrial cross-sectional area significantly declined with mid-piece length (untransformed model: estimated effect = −0.00223 µm^2^ µm^−1^, intercept = 0.147, *t* = −2.250, *p* = 0.027; *n* = 10 males, 10 area measures from each). The log-transformed data (figure 2*b*) showed a regression slope of −0.631 (95% CI = −1.087, −0.152) which is significantly different (*p* = 0.0324) from 0 but not from −1 (*p* = 0.1519). This was sufficient to keep mitochondrial volume roughly consistent across a range of mid-piece lengths (figure 2*c*). Volumes were computed using measured values of mitochondrial cross-sectional area and mid-piece length for 10 males, assuming mid-piece cross-sectional area is consistent within individual sperm (within male repeatability of single measures *R* = 0.075, extrapolated repeatability from 10 measures *R* = 0.44 [42]; i.e. helices do not taper; see Discussion).

The average radius of the mid-piece, as measured from the centre of the flagellum to the centre of the mid-piece helix, was 0.293 ± 0.05 µm (mean ± s.d.; *n* = 10 males, 10 measures from each male). A previous estimate of a diameter of 3 µm derived from scanning electron micrographs [43] was an error (T.R.B. 2018, personal observation). Our accurate radius measurements show a negative relationship with mid-piece length between males (estimated effect = −0.00274, intercept = 0.366, *t* = −2.519, *p* = 0.036; *n* = 10 males, 10 measures from each).

**Figure 3.**
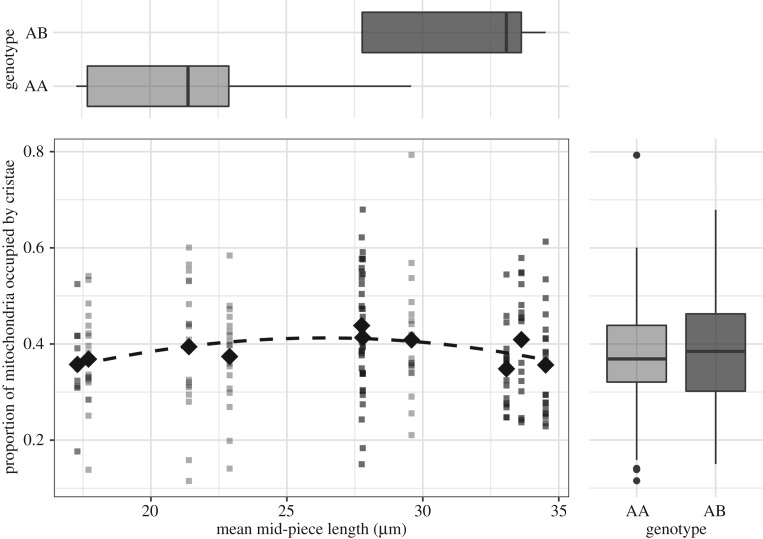
Mitochondrial packing shows a marginal but significant increase with mid-piece length up to 28 µm. Sperm trait genotype, which is a determinant of mid-piece length (boxplots top), did not affect mitochondrial packing (boxplots right). The data in light grey are from AA genotype males (*n* = 5, 20 measures each) and data in dark grey are from AB genotype males (*n* = 5, 20 measures each). The diamond-shaped points represent the mean values for every male.

The density of cristae was highly variable within males (figure 3) such that an individual's mean value from 20 measures reached an extrapolated repeatability of only *R* = 0.34 [42]. We found a statistically significant quadratic relationship between mid-piece length and cristae density (χ^2^ contrast to null model; *p* = 0.040, d.f. = 2, *n* = 10 males; figure 3), with a 15% difference between the highest density at mid-piece length of 27.8 µm and lowest density at mid-piece length of 17.3 µm.

## 4. Discussion

The results of this study show that sperm mitochondrial cross-sectional area decreases with mid-piece length in the zebra finch, meaning that mitochondrial volume remains relatively consistent across sperm with a wide range of mid-piece lengths (figure 2*c*).

We also observed large variation in cristae density within males (figure 3). This points to an inherent variability in cristae packing density in sperm cell mitochondria. This variation may be partly explained by the dynamic nature of mitochondria which regulate metabolism by rearranging their internal structure between two main states—a condensed matrix with wide cristae and a wide matrix with condensed cristae [44,45]. The triggers for, or the time period over which such rearrangement might take place is not known for sperm cells for any taxa. Despite this variation, mitochondrial packing density showed a small but significant quadratic relationship with mid-piece length suggesting that mid-sized mitochondria (length approx. 28 µm) may be most efficiently packed in zebra finch sperm.

Together, the above do not help explain the results of Bennison *et al.* [19], who found that sperm with shorter mid-pieces had greater ATP concentrations in zebra finches. Indeed, one interpretation of our results, which are based on a similar mid-piece length range as covered by this previous study [19], may be that energetic capacity does not vary with mid-piece length. However, we have shown that shorter mid-pieces have a larger mitochondrial cross-sectional area, which would translate to greater contact between the mitochondrial helix and flagellum due to its ellipsoid shape in cross section (figure 1*b*). OXPHOS in the mitochondria and glycolysis in the flagellum are strongly linked, with both pathways complementing and competing with each other to meet energetic demands [46]. It is likely that most bird species lie on a continuum between a reliance on OXPHOS versus glycolysis for sperm energy production [23,47,48]. Despite the absence of the fibrous sheath, passerine sperm may still be able to produce ATP through glycolysis, as many glycolytic enzymes can be localized unbound in the cytosol or bound to microtubules [21,49]. Analogues of some of the enzymes that bind to the fibrous sheath have been found associated with membrane domain proteins on the outer membrane of mitochondria in somatic cells [50,51]. Such interactions between the mitochondrial helix and flagellum, for the exchange of metabolites and metabolic products, could potentially increase metabolic efficiency, thereby offering an explanation for Bennison *et al.*'s [19] results. At the interspecific level, mid-piece length varies greatly within passerines, and it is perhaps unlikely that mid-piece volumes remain constant with length across this larger range. This may explain why the results of Bennison *et al.* [19] and Rowe *et al.* [18] do not agree.

Another possible function of the passerine mitochondrial helix is structural support for motility. The fibrous sheath plays this role in other sperm [52], indicating these structures may be functionally analogous. Although mid-piece length has no clear independent effect on swimming velocity [19], the mid-piece to tail length ratio does [19,38], providing evidence for subtle interactive effects of mid-piece length with other sperm traits [53]. All of this suggests an adaptive advantage to having a proportionately longer, if not larger mid-piece, at least in passerines.

An interesting and unexpected finding of this study was that zebra finch sperm are an order of magnitude thinner than previously reported (0.293 µm compared with 3 µm radius [43]). The mid-piece radius was negatively related to mid-piece length between males and the sperm mitochondrial helix had a regular helical periodicity, with a repeatable gyre height of 3.783 µm. Together, these results can be substituted into the general equation proposed by Birkhead *et al.* [43] for measuring the length of the mid-piece when unwound: 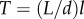; where 
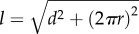
, *T* is the straight helix length, *L* the length of the mid-piece, *d* the gyre height and *r* the radius of the mid-piece helix. This equation can then be simplified to 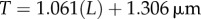 (from regression of straight helix length on mid-piece length).

It must be noted that we have assumed sperm mid-pieces to be cylindrical helices 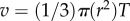, with a consistent radius along the mid-piece. Owing to insufficient resolution of SPIM and an absence of positional information with TEM, we could not test this assumption. However, by calculating the approximate volume of a truncated cone, one can see that the trade-off between volume and thickness would persist even if the mid-piece was tapered, irrespective of degree of tapering (electronic supplementary material, methods). Moreover, a tapered morphology has not been noted by previous studies, including those employing high-resolution electron microscopy [54,55].

Our study highlights a caution against using length as a proxy for size, especially when it comes to complex structures such as the passerine mid-piece. Length measures can still help explain sperm function but only in conjunction with methods that quantify interactions between the different sperm traits.

In conclusion, we have shown that the zebra finch sperm mid-piece gets thinner with increasing length, providing evidence of a trade-off between sperm thickness and length in a species with high levels of sperm morphological variation.

## Supplementary Material

Supplementary Methods

## Data accessibility

Datasets are available from Dryad Digital Repository (http://dx.doi.org/10.5061/dryad.2b549t4) [56].
