# Supplementary material for: A trade-off between thickness and length in the zebra finch sperm mid-piece
Source: Proc Biol Sci. 2018 Jul 25;285(1883):20180865. doi: 10.1098/rspb.2018.0865 (PMC6083248; doi:10.1098/rspb.2018.0865)
Supplement: Supplementary Methods [file rspb20180865supp1.docx]

**A trade-off between thickness and length in the zebra finch sperm mid-piece (Supplementary Methods)**

Tania Mendonca, Tim R Birkhead, Ashley J Cadby, Wolfgang Forstmeier, and Nicola Hemmings

**Part 1: Mid-piece structural organisation**

*Image acquisition and processing with selective plane illumination microscopy (SPIM)*

10 µL of sperm (n = 19 males) labelled with 500 µmol/L MitoTracker^TM^ Green FM (Molecular Probes, Eugene, OR) was mixed with blue-green fluorescent microbead fiducial markers (1 µm diameter, 1:800 dilution; Invitrogen^TM^) and suspended in agarose (low gelling point, final dilution of 1% (w/v); Sigma Aldrich). This mixture was aspirated into an OpenSPIM sample holder [1] - a 1 mL syringe modified to hold a shortened 20 µL capillary and plunger (Wiretrol^®^ I, Drummond Scientific Company), and left to set for 30 mins.

Agarose embedded sperm samples were prepared for each male and 200 µm deep image stacks of each imaging volume were acquired at 5 angles (0º, 72º, 144º, 216º and 288º) in PBS using a custom-built SPIM microscope (at the University of Sheffield) with laser excitation at 473 nm and a 525 nm (15) band pass (BP) fluorescence emission filter (Semrock, Inc.). The microscope hardware and optical components are based on the OpenSPIM platform [1] but modified to include alternative lasers (473 nm, 30 mW; 532 nm, 10 mW; and 640 nm, 110 mW), magnification at 28X and an alternative sample positioning system. The sample positioning system used included coarse positioning using three stepper motor actuators (NanoPZ series, Newport Corporation) and a rotation stage (M-RS65, Newport Corporation). Sample scanning was performed using a piezo motor nanostage system (PIMars P-562.2CD, Physik Instrumente Ltd) with a position sensor that triggered the camera at regular travel distances. Images were captured using a sCMOS camera (Orca Flash 4.0 V2 C11440-22CU, Hamamatsu Photonics) which was triggered by the piezo stage controller (E-725.3CD, Physik Instrumente Ltd) and files were written to disk using HCImageLive software (Hamamatsu Photonics). The camera, detection and illumination objectives, and magnification are fixed for the system, ensuring that the imaging results are highly reproduceable.

Multi-view registration and deconvolution was performed on the acquired image z-stacks using the ‘multi-view deconvolution’ plugin [2] in Fiji [3] to acquire a single deconvolved stack for each imaging volume. After multi-view deconvolution, the system point spread function (PSF), a measure of resolution limit, was 0.654 µm in the lateral direction and 1.22 µm in the axial direction. This was considered adequate at the time for making volumetric measurements from the deconvolved image stacks of zebra finch sperm as they have been previously estimated to have a radius of ~3 µm [4].

*Sperm mid-piece length (from SPIM)*

Region of interest (ROI) volumes were cropped around individual sperm in the image stacks in Fiji [3]. 3D length measurements of the mid-piece were acquired using the semi-automated plugin ‘Simple Neurite Tracer’ (measurement repeatability; R = 0.997) [5]. This plugin traced the helical shape of the mid-piece and therefore, measured the length of the mitochondrial helix if it was unwound and straightened (‘straight helix length’). The length of the mid-piece in its coiled state, along the flagellum, was computed by interpolating the straight helix length trace from the ‘Simple Neurite Tracer’ plugin in each axis using the ‘interp1’ function in MATLAB^®^ (2015b, version 8.6, The Math Works, Natick, MA) and is referred to simply as ‘mid-piece length’ in this study.

*Sperm mid-piece volume (from SPIM)*

For each deconvolved z-stack, regions of interest (ROIs) were drawn around individual sperm. For each sperm, a selection was made around the mid-piece to isolate the image volume containing the labelled mitochondrial helix and this was then binarised using a threshold. The threshold for each image volume was selected using the interquartile range rule [3Q + (1.5 x IQR)] where the outliers – the highest intensities in the image volume – belong to the signal from the mitochondrial helix. The volume was computed by counting the thresholded pixels using the ‘regionprops’ function from the Image Processing Toolbox in MATLAB and scaling this value to match the microscope voxel ratio (0.234 µm x 0.234 µm x 0.234 µm) after multi-view deconvolution.

**Part 2: Mid-piece internal organisation**

*Image acquisition and processing with transmission electron microscopy (TEM)*

The left SGs from each of the ten males used in Part 2 were incubated in a glass vial with standard fixative solution [2.5 % Glutaraldehyde (Sigma Aldrich), 4 % Formaldehyde EM grade (Science Services) and 100 mM Sodium Phosphate Buffer (Na_2_HPO_4_.2H_2_O and NaH_2_PO_4_.2H_2_O, Sigma Aldrich), pH 7.5] at room temperature for 2 hours with gentle agitation (100 rpm), in preparation for TEM imaging. After 2 hours, the glass vials with the SGs were transferred to a fridge at 4º C for a minimum of one and maximum of two days. At the end of fixation, the SGs were transferred into micro-centrifuge tubes containing cold sodium phosphate buffer at 4º C for transport to the University of York for further processing and TEM imaging.

The fixed SGs from the 10 males were embedded in resin blocks and 600 nm sections were cut for TEM imaging. These sections were negatively stained with uranyl acetate and lead citrate before being imaged using a Tecnai 12 TEM (FEI, OR) at 43000X. The TEM microscope uses electrons instead of visible light to image these sections. Since the wavelength of electrons (~0.004 nm at 100 keV) is much smaller than that of visible light (390 nm – 700 nm), the resolution of TEM is significantly better than brightfield or fluorescence microscopy.

*Sperm cristae density measurement*

In each image a ROI was defined around the mitochondrial section and this was binarised using a manual threshold for each image to isolate the mitochondrial matrix (thresholding was performed by TM throughout). The count of the thresholded pixels was calculated using the ‘regionprops’ function in MATLAB to give the area occupied by the mitochondrial matrix (Figure 1 (d)). The ‘imfill’ function in MATLAB was used to dilate the matrix selection to fill gaps occupied by the cristae (Figure 1 (e)). This gave a selection that described the area occupied by the mitochondria in cross-section. The area occupied by the cristae was derived by subtracting the area occupied by the matrix from the total area of the mitochondria in cross-section, i.e. area occupied by cristae = area in Figure 1 (d)– area in Figure 1 (e). Cristae density was measured as the proportion of the area occupied by cristae to the area of the mitochondria.

*Sperm mid-piece measurements*

In the TEM cross-sections, sperm mitochondria had an elliptical shape with the minor axis in-line with the flagellar diameter and the major axis extending to either side of the flagellum (Figure 1(b)). Measurements of (1) the flagellum diameter, (2) mitochondrial minor axis diameter, and (3) mitochondrial major axis diameter were made from 10 images from each male (n = 10 males) where the microtubule doublets were in a circular arrangement, indicating that the section was approximately 90º to the sperm longitudinal axis (Figure 1(b)).

**The trade-off between mid-piece volume and thickness persists irrespective of degree of tapering**

A rough calculation was performed assuming the volume of a truncated cone $v = \frac{1}{3}\pi(r_{1}^{2}+r_{1}r_{2}+r_{2}^{2})l$, where v is the volume of the mid-piece, r_1_ and r_2_ are the radii of the mitochondrial helix at either end of the mid-piece and l is the length of the mid-piece. r_1_ and r_2_ were calculated such that the mean of their values corresponded with predicted values from a model of the relationship between mitochondrial helix radius (the mean of half the measures ‘2’ and ‘3’ from Figure 1(b)) and mid-piece length, and the ratio between them was varied from 1 (no taper) to 10. Mid-pieces with larger r_1_ to r_2_ ratios have larger volumes overall but still show a trade-off with mid-piece length (Figure S1). The mitochondrial helix is ellipsoid in cross-section and therefore, the above formula overestimated the volume, however the relationship between volume and length followed roughly the same trend as found experimentally (Figure S1).

*Figure S1*


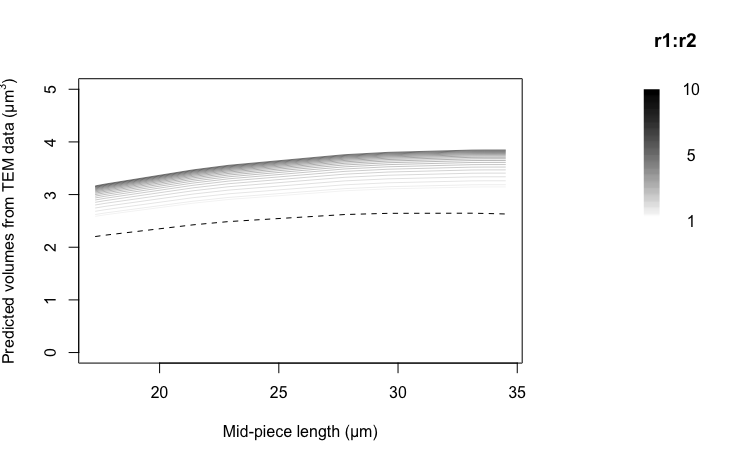


**Figure S1:** Trade-off between mid-piece volume and length assuming different degrees of tapering. The dotted line represents a fit to values from volumes calculated using TEM data (Part 2 of Results and Figure 2(c)). Above the dotted line, each line represents volumes calculated assuming a different r_1_ to r_2_ ratio (from 1 to 10, with increments of 0.5).

**References:**

1. Pitrone PG, Schindelin J, Stuyvenberg L, Preibisch S, Weber M, Eliceiri KW, Huisken J, Tomancak P. 2013 OpenSPIM: an open-access light-sheet microscopy platform. *Nat. Methods* **10**, 598–599. (doi:10.1038/nmeth.2507)

2. Preibisch S, Saalfeld S, Schindelin J, Tomancak P. 2010 Software for bead-based registration of selective plane illumination microscopy data. *Nat. Methods* **7**, 418–9. (doi:10.1038/nmeth0610-418)

3. Schindelin J *et al.* 2012 Fiji: an open-source platform for biological-image analysis. *Nat. Methods* **9**, 676–82. (doi:10.1038/nmeth.2019)

4. Birkhead TR, Pellatt JE, Brekke P, Yeates R, Castillo-Juarez H. 2005 Genetic effects on sperm design in the zebra finch. *Nature* **434**, 383–7. (doi:10.1038/nature03374)

5. Longair MH, Baker DA, Armstrong JD. 2011 Simple neurite tracer: Open source software for reconstruction, visualization and analysis of neuronal processes. *Bioinformatics* **27**, 2453–2454. (doi:10.1093/bioinformatics/btr390)
